# A high-speed brain-computer interface (BCI) using dry EEG electrodes

**DOI:** 10.1371/journal.pone.0172400

**Published:** 2017-02-22

**Authors:** Martin Spüler

**Affiliations:** Department of Computer Engineering, Eberhard-Karls University Tübingen, Tübingen, Germany; Shanghai Jiao Tong University, CHINA

## Abstract

Recently, brain-computer interfaces (BCIs) based on visual evoked potentials (VEPs) have been shown to achieve remarkable communication speeds. As they use electroencephalography (EEG) as non-invasive method for recording neural signals, the application of gel-based EEG is time-consuming and cumbersome. In order to achieve a more user-friendly system, this work explores the usability of dry EEG electrodes with a VEP-based BCI. While the results show a high variability between subjects, they also show that communication speeds of more than 100 bit/min are possible using dry EEG electrodes. To reduce performance variability and deal with the lower signal-to-noise ratio of the dry EEG electrodes, an averaging method and a dynamic stopping method were introduced to the BCI system. Those changes were shown to improve performance significantly, leading to an average classification accuracy of 76% with an average communication speed of 46 bit/min, which is equivalent to a writing speed of 8.8 error-free letters per minute. Although the BCI system works substantially better with gel-based EEG, dry EEG electrodes are more user-friendly and still allow high-speed BCI communication.

## Introduction

A Brain-Computer Interface (BCI) is a device that allows to control a computer by brain activity only, without the need for muscle control [1]. Its main purpose is to restore or improve communication for paralysed patients. While there are different kinds of BCI that either rely on motor imagery [2] or the detection of event-related potentials like the P300 [3], BCI systems based on visual evoked potentials (VEPs) are currently the fastest method to establish non-invasive BCI control [4, 5]. Using code-modulated visual evoked potentials (c-VEPs), it was shown that participants could spell more than 20 error-free characters per minute (> 140 bit/min) [4]. When using steady state visual evoked potentials (SSVEPs), the communication speed can further be improved by using frequency and phase information of the SSVEP to reach communication speeds up to 60 characters per minute (> 300 bit/min) [5].

While these results show that VEP-based BCIs allow for high-speed communication in a lab environment, there are only few efforts to transfer those systems out of the lab and make them useable for a broader audience. One of those few approaches is the *Tübingen c-VEP BCI*, which was previously shown to reach spelling speeds of more than 20 error-free letters per minute and in another work was improved to control mouse and keyboard of the Windows^®^ operating system, thereby enabling a user to completely control a computer and use arbitrary applications by means of his brain activity only [6]. While this is an important step towards improved useability of a BCI system, the use of gel-based EEG devices still limits the applicability, as gel-based EEG is time-consuming to apply, can not be applied by the user itself, and “few people are ready to wash their hair each time they wish to use Word or a LaTeX editor” [7].

To solve this problem, dry EEG electrodes have been developed that are quick to setup and allow to measure EEG without the need for conductive gel. While there are a variety of different dry electrodes being developed [8–15], there are also commercially available dry EEG electrodes that deliver good results and have been shown to work for BCI applications using P300, SSVEP and motor imagery [16, 17]. Further, there are consumer-grade EEG headsets with dry electrodes or easy to apply wet electrodes that don’t need the application of conductive gel like the Emotiv EPOC. While such systems have also been used for BCI applications [18], the signal quality of such EEG hardware is not comparable to professional devices and their use is not recommended for serious applications [19].

A good comparison of different dry EEG electrodes developed and tested can be found in Baek et al. [20] who review and discuss several publications. They conclude that dry electrodes allow the acquisition of spontaneous EEG signals with lower signal quality than gel-based EEG electrodes and that movement artifacts are a larger problem in dry electrodes. Nevertheless, signal quality is good enough to record P300 and SSVEP response to use them for BCI applications.

While dry electrodes haven’t been tested with a c-VEP BCI system so far, the aim of this study was to test if commercially available medical-grade dry EEG electrodes can be used with a high-speed c-VEP BCI and what algorithmic improvements of the system are necessary to deal with the increased signal-to-noise ratio of dry EEG electrodes.

## Methods

### c-VEP Brain-Computer Interface

**Fig 1:**
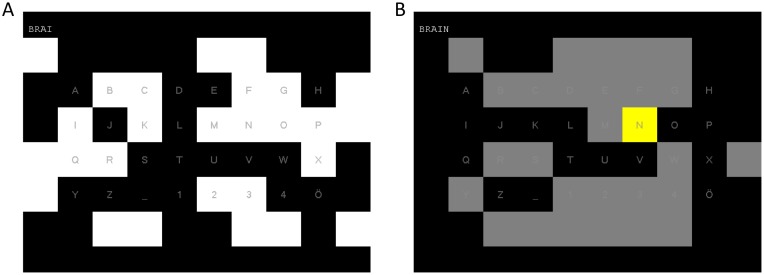
Screenshot of the c-VEP BCI. **A:** screenshot during a trial. The letter in the lower right corner serves as a backspace symbol during the free-spelling condition and allows the user to correct mistakes. **B:** screenshot of the letter N being selected and the other characters being grayed out to indicate a selection.

The c-VEP BCI system used in this study is the one presented in a previous publication [4], where it is described in more detail. With this system, 32 target stimuli are displayed on a computer screen, which are modulated with a pseudo-random code and allow the user to select one of 32 characters. The 32 targets are arranged as a 4 × 8 matrix and 28 complementary non-target stimuli are surrounding the targets (Fig 1 shows a screenshot of the stimuli as presented to the user). For modulation of the stimuli, the same pseudo-random code was used for all stimuli, but was shifted 2 bit for each stimulus, resulting in a time lag *τ*_*s*_ = 2/60 = 0.033 s between two consecutive targets. If the user focuses on a stimulus, the changes in luminance, based on the pseudo-random code, evoke a potential with a special shape that can be observed in the EEG, the so called code-modulated visual evoked potential (c-VEP). Based on the time lag of the c-VEP, it can be detected which stimulus the user is focusing on and thereby which character the user wants to select.

#### EEG recordings

EEG was recorded with a g.tec g.USBamp amplifier (g.tec, Graz) at a sampling rate of 600 Hz. A notch filter at 50 Hz and a bandpass filter between 0.5 Hz and 60 Hz was applied during recording. 15 g.Sahara electrodes (g.tec, Graz) were used, which are active dry electrodes, each consisting of 8 pins made of a special golden alloy. The electrodes were placed according to the 10–20 system (Fz, T7, C3, Cz, C4, T8, CP3, CPz, CP4, Pz, PO3, POz, PO4, O1, O2) and referenced to a dry electrode at Oz. The ground electrode was placed at the right mastoid. As the dry EEG electrodes do not support impedance measurement, impedance was not checked. But the signal was inspected visually and electrodes that appeared to be very noisy were moved around a little, to remove hair between electrode and skin and thereby get better contact of the electrodes.

#### Processing and classification of EEG signals

To deal with the different noise floor of the dry electrodes, a zero-phase FIR bandpass filter with the range of 1 Hz to 30 Hz was applied to the data. For classification of the c-VEP, a spatial filter using canonical correlation analysis (CCA) was constructed [21] and a template of the c-VEP was constructed by averaging multiple trials. By shifting the template, according to the time lag of the target stimuli, templates for all targets were obtained. To detect which target the user was focusing on, the Pearson correlation between the recorded EEG and all templates was calculated and the target with the highest correlation was chosen. Although a different method using a one-class support vector machine (OCSVM) and the euclidean distance showed better classification results than using correlation [22], correlation has the benefit of delivering a normalized output in the range [-1, 1], which is an important feature that was needed for the dynamic stopping method, which will be introduced in the next section.

To train the system, a co-adaptive calibration process was used (as in [4]), in which the participants where given 64 characters to write and they received immediate feedback starting from the first letter. After the calibration phase, adaptation was turned off and the system was tested with a fixed classifier.

#### Averaging and dynamic stopping

While the c-VEP BCI with gel-based EEG used a 1.05 s window of EEG data to classify the user’s intent, it was expected that this might not be enough data when using the c-VEP BCI with dry electrodes, due to the lower signal-to-noise ratio of these electrodes. Therefore, an averaging method was used, in which multiple trials (called epochs in this context) are presented in a row and the signal is averaged over multiple epochs before classification. Such an averaging method is also used in the popular P300 BCI [3] and allows high classification accuracies even with a bad signal-to-noise-ratio. In this study, different number of epochs to average were tested ranging from 1 to 3 epochs.

Further, we introduced a dynamic stopping method that collects epochs until a certain stopping criterion is reached. Such dynamic stopping methods were previously used in P300 [23, 24] and SSVEP BCIs [25]. After each epoch, the average of the collected epochs is calculated and the data is classified by correlating the data with the templates. If one of the correlations is > 0.4, the trial stops and the corresponding target is selected. Otherwise, more data is collected until the stopping criterion is reached. As the averaging over multiple epochs improves the signal-to-noise-ratio of the data, the dynamic stopping method should ensure that enough data is collected to ensure a high quality of the prediction.

As mentioned above, correlation was used in this work as it is better suited for dynamic stopping. Using OCSVM would result in a distance value for each template. As the distance values depend on the data of each subject, the range in which they are distributed varies, which makes it difficult to find an appropriate threshold as it is different for each subject. On the other hand, correlation delivers a normalized output in the range [-1, 1], thereby making it possible to choose one threshold which works for all subjects.

### Study design and participants

For this study, 12 participants were recruited, who all were students at the university. They were either paid for participation in this study (8 € per hour) or received credits relevant for their studies. All subjects had normal or corrected-to-normal vision and no previous BCI experience. The study was approved by the local ethics committee of the Medical Faculty at the University of Tübingen and written consent was obtained from all participants.

For each subject, the experiment was divided into 4 blocks, which were separated by short breaks. In each block, one of the 4 conditions (averaging of 1, 2, 3 epochs, and dynamic stopping) was tested. One block consisted of 3 runs, with each run consisting of 64 trials. In the first run, the system was calibrated in a co-adaptive manner, in which the participants received feedback from the BCI during the whole calibration process. After the first run, the classifier and spatial filter did not change and to evaluate the performance of the system, it was tested in 2 runs, which is a total of 128 trials (letters). For the 3 blocks using 1, 2, or 3 epochs for averaging, the same settings (number of epochs to average) was also used for the training. Thereby, the total time needed for calibration of the system was 125, 192, and 259 seconds, respectively. For the dynamic stopping condition, the system was calibrated using 3 epochs to average, but tested afterwards with dynamic stopping.

To ensure that results are not confounded by non-stationary effects over time, the order, in which the 4 blocks were presented, was permuted for each subject. Thereby it was ensured that the occurrence of each block was distributed equally over time.

As block 4 used the dynamic stopping approach, there was a possibility that the stopping criterion would be very hard to reach in case of a bad signal-to-noise-ratio, potentially resulting in a very long time needed for one trial. Therefore, block 4 was prematurely terminated if the first 32 testing trials took longer than 10 minutes, which was the case for 2 subjects (S06 and S12).

### Evaluation

To evaluate the performance of the c-VEP BCI with dry electrodes and compare the different methods, accuracy and bitrate were used as measures. Accuracy is the percentage of correctly selected letters, while the bitrate is based on the information transfer rate (ITR) proposed by Wolpaw et al. [26]. The ITR giving the information per trial (bit/trial) with N being the number of classes (N = 32 in this study) and P being the accuracy is defined by:

$$ITR\left(N,P\right)=\log_{2}N+P\cdot \log_{2}P+\left(1-P\right)\log_{2}\left[(1-P)/(N-1)\right]$$ (1)

Dividing the ITR by the time in seconds needed to select one letter (including inter-trial-interval) yields the bitrate in bit/sec. While the ITR is a widely used metric to assess the performance of BCIs, it is problematic as it should only be used when certain criteria are met [27]. Further, it represents a theoretical upper bound of how much information can theoretically be transmitted with this BCI using an optimal encoding strategy. As a user won’t be able to use such optimal encoding strategies, but rather select the desired letters and delete errors with a backspace-command, the ITR is not a good indicator of the real performance. To assess the real transferred information, measures like utility metric [28] or equivalent approaches [29] can be used, which take into account that every wrong letter has to be corrected by selecting a delete-command. Based on this idea, we calculated the average correct letters per minute (CLM) which is given by

$$CLM\left(P,T\right)=\left\{\begin{array}{cc} \frac{60\cdot (2P-1)}{T} & \text{if}\;P\geq 0.5\\ 0 & \text{otherwise} \end{array}\right.$$ (2)

where T is the average time in seconds for one trial and P the accuracy. As this metric delivers an exact number of how many letters a user can write per minute, taking into account that all errors are corrected by the user, so that the written text contains only error-free letters, this metric is a more direct measure of the BCI performance as experienced by the user.

To illustrate the problems of ITR to assess real-life performance and the benefits of CLM, a short example is given. Assume the c-VEP BCI has two options to use either 1 sec. of data to achieve 29% accuracy or 19 sec. of data to achieve 100% accuracy (with 1 sec. break between two trials). This performance would translate to a bitrate of 18 bit/min for the 1 sec. option and 16 bit/min for the 19 sec. option, which would lead to the decision to use the 1 sec. option. However, with 29% accuracy the system is not useable at all, while it is slow but perfectly useable with the 19 sec. option. Looking at the CLM, the 1 sec. option would results in 0 CLM, while the 19 sec. option would result in 3 CLM. Thereby, the CLM clearly reflects the usability of a system, while the bitrate does not.

### Comparison with gel-based EEG study

To compare the results using dry EEG electrodes with results using gel-based EEG electrodes, we used data from a previous study [4], in which 9 subjects used the c-VEP BCI with 32 gel-based EEG electrodes (Acticap, Brainproducts). The c-VEP BCI was calibrated first using 64 trials (1 epoch) and afterwards tested with 576 trials using 1 epoch. For online classification, an OCSVM was used with an ErrP-based online adaptation [4]. As the number of electrodes and the method of classification were different in the online gel-based EEG study, results can not directly be compared. Therefore, we performed an offline simulation of an online experiment with the gel-based EEG data using only 15 electrodes (same positions as for the dry electrodes) and the same classification method (with 1 epoch) as was used with the dry EEG.

## Results

The main results of the study is that the c-VEP BCI with dry electrodes performed best using the dynamic stopping approach with an average accuracy of 75.9%. With an average time per trial of 13.63 s, this corresponds to a bitrate of 46.2 bit/min or 8.76 correct letters per minute. Detailed results for the accuracy can be found in Table 1, for the bitrate in Table 2 and in Table 3 for the correct letters per minute.

**Table 1:** Accuracies for the c-VEP BCI with dry EEG electrodes.

	fixed stopping			dynamic stopping	
Subject	1 epoch	2 epochs	3 epochs	Accuracy	time per trial
S01	82.8%	92.2%	80.5%	82.0%	2.93 s
S02	70.3%	93.8%	83.6%	96.1%	3.02 s
S03	7.8%	40.6%	43.8%	85.9%	7.34 s
S04	28.9%	34.4%	84.4%	83.6%	4.94 s
S05	73.4%	93.8%	100.0%	94.5%	3.69 s
S06	2.3%	8.6%	3.1%	12.5%	77.00 s
S07	57.8%	74.2%	79.7%	89.8%	3.83 s
S08	62.5%	96.9%	53.1%	99.2%	3.38 s
S09	18.8%	59.4%	53.9%	69.5%	5.26 s
S10	15.6%	39.8%	28.1%	55.5%	6.94 s
S11	50.8%	60.2%	32.8%	87.5%	4.66 s
S12	6.3%	12.5%	28.1%	54.2%	40.58 s
mean	39.8%	58.9%	55.9%	75.9%	13.63 s

Shown is the average accuracy for each subject, as well as the mean accuracy over all subjects, when using different number of epochs to average. For the dynamic stopping, the average time (including break) needed to write one character is shown. The time needed for one character (including break) with the fixed stopping method is 1.96 s with 1 epoch, 3.02 s with 2 epochs and 4.07 s with 3 epochs.

**Table 2:** Average bitrate for the c-VEP BCI with dry EEG electrodes.

	fixed stopping			dynamic stopping
Subject	1 epoch	2 epochs	3 epochs	
S01	106.5	84.3	48.9	70.3
S02	82.0	87.0	52.4	91.3
S03	1.2	21.9	18.3	30.5
S04	18.5	16.5	52.7	43.1
S05	87.9	87.3	74.0	71.9
S06	0.1	1.3	0.3	0.1
S07	59.3	58.1	48.4	63.0
S08	67.4	93.0	34.0	87.2
S09	8.7	39.9	25.3	29.8
S10	6.0	20.8	8.6	15.6
S11	47.0	40.7	11.2	49.4
S12	0.7	2.6	9.5	2.6
mean	40.4	46.1	32.0	46.2

Shown is the average information transfer rate (ITR) in bit/min for each subject with the different methods, as well as the average over all subjects.

**Table 3:** Average correct letters per minute (CLM) for the c-VEP BCI with dry EEG electrodes.

	fixed stopping			dynamic stopping
Subject	1 epoch	2 epochs	3 epochs	
S01	19.89	16.70	8.95	13.10
S02	12.50	17.49	9.93	18.34
S03	0	0	0	5.87
S04	0	0	10.05	8.17
S05	14.41	17.50	14.81	14.48
S06	0	0	0	0
S07	4.81	9.68	8.79	12.47
S08	7.69	18.75	0.92	17.50
S09	0	3.71	1.15	4.46
S10	0	0	0	0.95
S11	0.48	4.01	0	9.66
S12	0	0	0	0.12
mean	4.98	7.32	4.55	8.76

Shown are the average correct letters per minute (CLM) for each subject with the different methods, as well as the average over all subjects.

Comparing the dynamic stopping method to the fixed stopping, the dynamic stopping performed better with all performance metrics (Accuracy, bitrate, CLM). While the dynamic stopping method showed a significantly higher accuracy than all fixed stopping methods (Wilcoxons test, *p* < 0.05), there was no significant difference between the methods regarding the bitrate (Wilcoxons test, *p* > 0.05). The average correct letters per minute (CLM) using dynamic stopping was higher than for the fixed stopping using 1 or 3 epochs (Wilcoxons test, *p* < 0.05), but the difference to the fixed stopping with 2 epochs was not significant (*p* > 0.05).

While accuracy and bitrate are more commonly used metrics, they are of lesser importance to the user, which is why we evaluated the system also in correct letters per minute, as a user-centered metric. With dynamic stopping, only 1 out of 12 subjects had an accuracy lower than 50% and a CPM of 0. While classification accuracy was still above chance level, it was not good enough to enable BCI control for those subjects. With the fixed stopping, the number of subjects who couldn’t control the BCI was much higher, with 6 out of 12 having a CPM of 0 in the fixed stopping condition and 5 out of 12 subjects having a CPM of 0 with fixed stopping and 2 or 3 averaged epochs.

### Comparison with gel-based EEG study

The results from the comparison of gel-based EEG with dry EEG electrodes are shown in Table 4. When comparing the results from the gel-based EEG data with 15 electrodes, accuracy, bitrate and CLM are significantly higher than for the dry EEG (*p* < 0.005, Wilcoxon’s ranksum test).

**Table 4:** Comparison of dry EEG with gel-based EEG.

	Accuracy	bitrate	CLM
dry EEG (online, 15 elec.)	75.87%	46.2	8.8
gel EEG (offline, 15 elec.)	83.69%	112.3	20.7
gel EEG (online, 32 elec.)	96.18%	144.0	28.4

Average accuracy, bitrate and correct letters per minute (CLM) for gel-based and dry EEG electrodes.

### Analysis of c-VEP waveform

**Fig 2:**
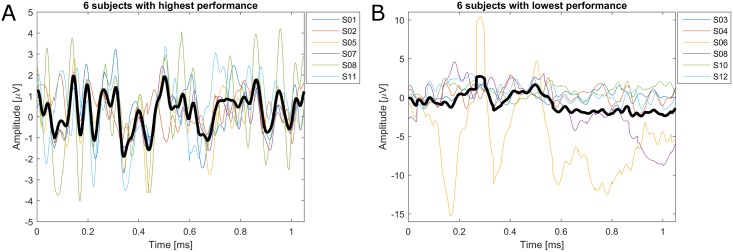
Average c-VEP waveform from the individual electrode where the c-VEP was strongest. Average over subjects is shown as bold black line and the individual subjects are shown by colored lines. **A:** for the 6 subjects with the highest performance **B:** for the 6 subjects with the lowest performance.

To investigate possible reasons, why the c-VEP BCI with dry electrodes works well for some subjects, while it does not for others, the c-VEP waveform was analyzed. Therefore, an offline analysis was performed to estimate classification accuracy using only a single channel. The channel with the highest classification performance for each subject was assumed to be the one where the c-VEP was strongest. Subjects were split into two groups depending on their classification performance. Average and individual c-VEPs are shown in Fig 2. While the group with the high performance shows a clear c-VEP waveform, such a c-VEP is not visible in the group having a low performance. In the low performance group, subjects S06 and S09 stand out as their average c-VEP shows clear signs of artifact contamination.

## Discussion

As the c-VEP BCI has previously been shown to achieve very high communication speeds with gel-based EEG electrodes [4], this work evaluated the performance of the c-VEP BCI using commercially available dry EEG electrodes. To cope with the worse signal-to-noise ratio expected with dry electrodes, changes in the signal processing chain were made and most importantly an averaging method and a dynamic stopping method were introduced to deal with the more noisy signals.

In a previous study using gel-based EEG electrodes [4], the subjects reached an average accuracy of 96%, which corresponds to 144 bit/min and an average of 28.4 correct letters per minute. In the here presented study, using dry EEG electrodes, an average accuracy of 76%, corresponding to 46.2 bit/min is reported which translates to an average of 8.76 correct letters per minute. As the results of the online study using dry electrodes are not directly comparable to the online results of the gel-based EEG study, an offline analysis was performed on the gel-based EEG data to evaluate performance using the same number and positions of electrodes and same methods for classification as for the dry EEG study. Performance dropped to an accuracy of 84%, which corresponds to a bitrate of 112 bit/min or 20.7 correct letters per minute. When comparing these results to the performance with dry EEG electrodes, the performance of the dry electrodes is significantly lower.

However, there seems to be a large inter-subject variance in the performance. For 1 out of 12 subjects the BCI did not work and had an accuracy below 50%, thereby leading to 0 correct letters per minute. 3 other subjects could use the system but had accuracies below 70% which is considered as threshold for satisfactory BCI communication [30]. On the other hand, 8 out of 12 subjects reached accuracies above 80%, which enabled those subjects to write with an average of 12.4 correct letters per minute. One subject even reached a communication speed of 19.9 correct letters per minute (or 106.5 bit/min). While the results using the dry EEG electrodes are significantly lower than with gel-based EEG, this still shows that the c-VEP BCI can be used with dry EEG electrodes for high-speed BCI communication.

Regarding the comparison of the different averaging and stopping methods tested in this study, the dynamic stopping method achieved the best results. For the fixed stopping condition, results were best when averaging 2 epochs, which is unexpected as one would assume to have a better signal-to-noise ratio when averaging over more data (i.e. using 3 epochs). One explanation could be that the longer trial length with 3 epochs led to a disengagement effect that negatively affected the BCI performance. However, even if an average of 2 epochs was the best setting for the fixed stopping condition, 5 out of 12 subjects could not communicate (0 correct letters per minute) and the accuracy and CLM were significantly lower than for the dynamic stopping method. These results show that it is not the best option to use the same BCI methods when switching from gel-based to dry EEG electrodes, but it is important to change the BCI algorithms to cope with the increased noise of the dry EEG electrodes. If such methods are implemented, performance can be significantly increased and thereby allow a broader userbase to successfully use the BCI.

Regarding the fact, that the classification accuracy was not high enough for some subjects to enable BCI control, it raises the question of what the causes are for the low performance. In motor imagery BCIs, there is a phenomenon called BCI illiteracy [31], which describes the fact that 15% to 30% of the users are not able to control a motor imagery BCI. Such a phenomenon could also be accountable for the effect seen in this study. However, in previous c-VEP BCI studies with gel-based EEG electrodes, such an effect was never reported and it worked well with all subjects (only one exception is reported due to the subject having trouble with its contact lenses [4]). To further investigate this issue, it is recommended for future studies to compare dry and gel-based EEG in the same subjects to verify if the effect found in this study is related to the dry electrodes.

The analysis of the c-VEP waveform shows that for the subjects with high performance, the c-VEP waveform is very similar to the one obtained in the gel-based EEG study [4]. For the subjects with low performance, the c-VEP is not clearly visible and there are signs of artifact contamination for 2 subjects. As dry EEG electrodes are very sensitive to movement artifacts, this is a likely explanation for low performance. For the other subjects with low performance, the c-VEP is also less prominent, which is likely due to a bad signal-to-noise ratio of the dry electrodes. The reasons are unknown, why there is a strong inter-subject variability in the signal-to-noise ratio. One possible explanation may be found in the physical properties of the subjects (very thick hair, use of hair products), but this point remains speculation as such properties were not systematically evaluated in this study.

## Conclusion

The c-VEP BCI has previously been shown to reach very high communication speeds with gel-based EEG electrodes. As users of the BCI system could profit from the increased useability of dry EEG electrodes, this study evaluates the performance of the BCI system with dry EEG electrodes. To deal with the lower signal-to-noise-ratio of the dry electrodes, different methods were tested and a dynamic stopping method showed the best results which enabled the subjects to write an average of 8.76 error-free letters per minute. While the performance of the BCI system with dry electrodes shows a high variability between subjects, it also shows that high-speed BCI communication with dry electrodes is possible and that bitrates > 100 bit/min can be achieved.
